# Introduction to the EC’s Marie Curie Initial Training Network Project: The European Training Network in Digital Medical Imaging for Radiotherapy (ENTERVISION)

**DOI:** 10.3389/fonc.2015.00265

**Published:** 2015-12-03

**Authors:** Manjit Dosanjh, Manuela Cirilli, Sparsh Navin

**Affiliations:** ^1^CERN, Geneva, Switzerland

**Keywords:** imaging, training, real time, hadron therapy, proton therapy, radiotherapy

## Abstract

Between 2011 and 2015, the ENTERVISION Marie Curie Initial Training Network has been training 15 young researchers from a variety of backgrounds on topics ranging from in-beam Positron Emission Tomography or Single Particle Tomography techniques, to adaptive treatment planning, optical imaging, Monte Carlo simulations and biological phantom design. This article covers the main research activities, as well as the training scheme implemented by the participating institutes, which included academia, research, and industry.

## Introduction

Cancer is a major societal issue, and by 2030 its global incidence is expected to increase by more than 75% in developed countries and by more than 90% in developing countries (1). A major challenge for cancer therapy is the complex and multifaceted nature of the disease, which calls for personalized treatments and an ever-expanding set of approaches in the oncologists’ toolbox. Radiotherapy (RT) has been used to treat tumors for more than a century, and still plays a major role in oncology: today, 50% of cancer patients receive RT, half of them with curative intent and is second only to surgery as a primary cure. At present, the mainstay of RT is photon therapy: this has become highly sophisticated, with methods like image-guided RT, intensity-modulated RT, stereotactic radiosurgery.

Despite the technological advances in RT approaches, the underlying dose deposition mechanism will always be the same: for photons, the deposited energy falls off exponentially as the photon beam traverses the body (except in the case of broad beams since scattering produces a departure of the attenuation from the exponential behavior). This makes it difficult to protect neighboring healthy tissues during treatment, which is an issue for deep lying tumors, tumors in/near critical organs, and pediatric tumors.

This is why RT with protons and other ions, known as Hadron Therapy (HT), has been proposed: in this case, most of the energy of the therapeutic beam is deposited at the end of its range in a characteristically peaked distribution (the Bragg peak), sparing the healthy tissue on the way to and beyond the tumor target.

The use of highly conformal dose distributions to improve the clinical outcomes of RT can be a double-edged sword. First, the target volume definition must be extremely accurate: if this is not the case, some tumor regions will not only receive a lower dose, as it also happens in RT with photons, but might not be irradiated at all, due to the steep dose gradients with protons and other ions. Temporal anatomic variations and organ motion have a more significant adverse influence on dose distributions in HT compared to RT with photons, making advanced imaging techniques a prerequisite for successful HT.

Independent studies carried out in Austria, France, Germany, Italy, and Sweden under the umbrella of the European Network for Light Ion Hadron Therapy (ENLIGHT) (2) provided evidence that 10–20% of RT cases may benefit from HT (3): these were conservative estimates, and therefore the actual numbers could be even higher.

While it is clear that photons will remain the backbone of RT, it is timely that the superior dose profiles of protons and carbon ions are fully exploited in clinical practice. Besides the need for clinical trials, the scientific community has strongly advocated for technology developments that would bring the current HT technology to the high standards of modern photon therapy.

Medical imaging is a key area to ensure the full exploitation of the potential of HT, in particular through quality assurance during treatment. Moreover, as new treatment centers are opening throughout Europe, there is an increasing demand for qualified experts in the multidisciplinary domains connected to HT. These issues were addressed by the ENTERVISION training project, a Marie Curie Initial Training Network aimed at educating young researchers in online 3D digital imaging for HT.

## The European Training Network in Digital Medical Imaging for Radiotherapy (Entervision)

**Figure 1:**
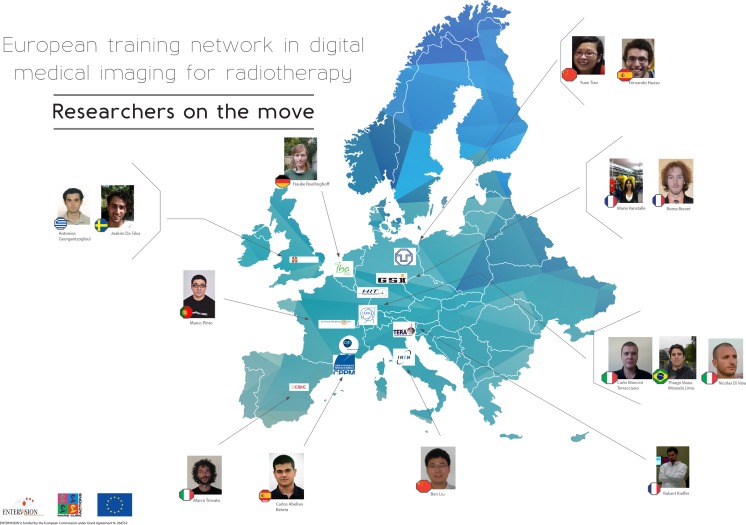
**Distribution of ENTERVISION researchers per nationality and recruiting institute**.

The ENTERVISION Marie Curie Initial Training Network was funded by the European Commission (EC) and launched in 2011, with the aim of educating young researchers in advanced medical imaging techniques for quality assurance of HT. Ten academic institutes and research centers of excellence, and a leading European company in HT (see Table 1), recruited 15 researchers from a variety of academic backgrounds over the course of 4 years (see Figure 1).

**Table 1:** **ENTERVISION partners: this table lists the Institutes, Universities, and companies participating to ENTERVISION**.

Acronym	Full name	Country
CERN	European Organization for Nuclear Research	Switzerland
CNRS	Centre National pour la Recherche Scientifique	France
GSI	GSI Helmholtzzen-trum für Schwer-ionenforschung GmbH	Germany
CSIC	Agencia Estatal Consejo Superior de Investigaciones Científicas	Spain
INFN	Istituto Nazionale di Fisica Nucleare	Italy
UCAM	University of Cambridge	UK
TUD	Technische Universität Dresden	Germany
TERA	TERA Foundation	Italy
IBA	Ion Beam Applications	Belgium
UCBL	Université Claude Bernard Lyon 1	France
UKL-HD	Universitätsklinikum Heidelberg	Germany

The ENTERVISION researchers were assigned individual research projects on topics ranging from in-beam Positron Emission Tomography (PET) or Single Particle Tomography techniques to adaptive treatment planning, optical imaging, Monte Carlo (MC) simulations, and biological phantom design. The majority of the researchers were also enrolled in a PhD program at a partner University, and a personalized career development plan was established by their supervisors for each researcher. In addition, the researchers took part in the network-wide training organized several times a year, offering a diversified portfolio of scientific courses, complemented by specific courses aimed at developing soft skills such as leadership and CV writing.

A unique feature of the ENTERVISION project was its connection with the EC-funded R&D project ENVISION, aimed at developing solutions for quantitative real-time non-invasive monitoring of HT for stationary and moving organs, accurate determination of delivered dose, and fast feedback to the Treatment Planning System (TPS) for optimal adaptation strategies. In fact, ENVISION acted as a “hands-on” training platform for the Marie Curie researchers, who had the opportunity to interact directly with senior scientists working at the forefront of research in quality assurance for HT.

The ENTERVISION researchers also benefited from the involvement in the ENLIGHT network. Throughout the project, the trainees have been encouraged to build a multidisciplinary network: this will not only help them with their future careers, but will ultimately improve the transfer of knowledge and collaboration between the various disciplines of cancer treatment.

### Detailed Research Program of ENTERVISION

The superior dose distribution of protons and other ions with respect to photons can be a double-edged sword if a series of factors (target volume definition, anatomical variations, organ motion) are not accurately determined and taken into account. A three-dimensional non-invasive imaging technique for real-time monitoring of the delivered dose is highly desirable, and several efforts toward this goal have been pursued within ENTERVISION.

At present, the most advanced method for HT quality assurance is PET. The use of heavy scintillating crystals coupled to silicon photomultipliers (SiPM) is one of the most promising solutions for future PET scanners. Developments in the field of particle detectors are focused on the use of time of flight (TOF) information that aims to improve the sensitivity by improving the signal to noise ratio.

ENTERVISION tackled the development of a characterization chain to measure the rising time profiles of signals in scintillating crystals used both for PET and high-energy physics (4). A rise-time measurement bench has been set up, where crystals are irradiated with a 511 keV gamma source, and the light produced is detected by a photomultiplier. In order to investigate the effect of thermalization inside the lattice, excitations at two lower energies are also foreseen. A pulsed X-ray machine excites the crystal with 20 keV photon pulses, and scintillation photons are collected with a streak camera system. The crystals are also exposed to 20 eV excitation energies at a vacuum ultraviolet (VUV) laser driven facility. This measurement chain allows complete access to thermalization lengths. Simulations in Geant4 (5) drive the choice of interesting crystal samples and set-up geometries.

Alternative detector choices have also been explored. One of the ENTERVISION researchers built a TOF–PET demonstrator with Multigap Resistive Plate Chambers (MRPCs) (6), achieving a preliminary time resolution of 240 ps sigma, and worked on a proton range telescope, developing an FPGA firmware to allow high rate acquisition (one million event per second) (7). They collaborated with another ENTERVISION researcher in order to prove the feasibility of distributing clocks over a MicroTCA-based optical fiber network, in order to synchronize electronic front-end boards at the pico-second scale. This would allow to perform TOF over a large-scale system dedicated to in-beam PET.

One of the challenges in using PET for HT monitoring, is to evaluate the motion-influenced artifacts. Within the framework of ENTERVISION, the influence of various motion parameters (peakiness, the ratio of inspiration and expiration, frequency, amplitude, drift, and parameter combination) was investigated through 40 experiments with radioactive sources performed at the GSI in-beam PET installation. 4D PET images were reconstructed, compared, and evaluated. The lateral field position and the particle deposition depth were studied with irradiated phantom experiments. PET artifacts caused by special respiratory motion cases (e.g., larger peak to peak amplitude) were also investigated. A potential artifact-compensation method was proposed, and a preliminary trial was conducted (8).

Single-particle imaging, i.e., detection of prompt photons, protons, or neutrons also resulting from nuclear interactions in the tissues, is emerging as a promising modality for dose monitoring during HT. ENTERVISION focused on improving prompt photon detection in the clinical scenario, through the development and test of gamma cameras, with both passive and active collimation systems.

One of the research projects carried out detailed comparisons between a multi-parallel-slit and a knife-edge slit collimator configuration (9). Detailed MC simulations allowed the setting of guidelines for choosing the optimal configuration of both camera types for various trade-offs between efficiency and spatial resolution. Measurements with a dedicated detector concept demonstrated, for the first time the capability of acquiring images at full clinical beam current, and further validated the results of simulation. Prototypes for both collimator types have been built and tested.

Active collimation systems (Compton cameras) have been also explored in depth. The ENTERVISION researchers assembled and tested a variety of detector geometries, materials, and read-out schemes. One of these is a three-layer Compton telescope based on continuous LaBr3 crystals and SiPM. The third layer has been completed recently, and included a new type of SiPM to increase the active area. The larger active area and a specific bias operating voltage for a single SiPM array brought an improvement of the energy resolution (10).

In parallel, a Compton camera has been developed and extensively tested in various beam conditions. Lutetium oxyorthosilicate (LSO) and bismuth germinate (BGO) commercial PET block detectors have been intensively tested and analyzed at different accelerators, in order to compare their performance and choose the absorber material. A considerable effort was made to improve the robustness and speed of the multi-threaded custom data acquisition system (DAQ) and to develop a platform for fast analysis. A prompt gamma-ray timing method for *in vivo* range verification has been proposed and tested at a clinical proton therapy facility, showing the great potential of this timing technique, with low footprint and cost and fast range retrieval (11).

In this variegated detector landscape, one ENTERVISION project aimed at developing a multi-purpose DAQ suitable for different medical imaging set-ups. The mezzanine boards work flawlessly, and the firmware is finished, tested and working. This firmware is intended to serve as a framework for detector developers, providing all the necessary tools to implement a full-featured DAQ without dealing with the board’s complexity, but by just writing the specific application VHDL and C code needed. Compatibility at a physics level has been verified with different read-out boards, while firmware-level compatibility is undergoing. In its current state, the DAQ system can be used in many different scenarios, from simple demonstrators to full featured imaging systems (12).

Highly realistic calculation models and fast simulation codes are required for most of these quality assurance tools. The high sensitivity of HT to motion and changes in patient anatomy calls for adaptive treatment delivery, where the delivered dose is actively monitored. Fast dose calculation, specifically recalculation of an existing treatment plan in modified anatomies, constitutes a crucial component in such a system. Also, the interaction of the incoming therapeutic beam with human tissues leads to the production of nuclear fragments and secondary light particles; hence, an accurate estimate of the dose deposited in the cancerous and healthy tissues requires sophisticated simulation tools based on nuclear reaction models. The validity of such models has to be assessed through extensive comparisons with as many sets of experimental data as possible.

One of the ENTERVISION research projects (13) focused on improving the nuclear models for carbon ion break-up. In particular, the researcher had the opportunity to work in collaboration with iThemba LABS where an experiment (14) with 33 MeV/n 12C ions on C, Au, Nb, and Polyethylene targets has been carried out. This experiment is the only one that took data studying in correlation all the fragments produced by the quasi elastic breakup of 12C in 8Be and 4He. Studying exclusively such a process is of particular interest because many experiments showed a broad peak in the 4He production with an energy per nucleon close to the beam energy. Moreover, as the 8Be decay almost immediately in two 4He, this is the only way to disentangle the He4 produced directly from 12C and from Be8 as intermediate state. Additionally, such a unique study sets a robust benchmark for future models and MC simulations. Unusual features in the energy distributions of the fragments suggest an H contamination of the targets, a hypothesis confirmed by a second experiment with a polyethylene target. The contribution of H contaminants to carbon break-up experiments has been studied, modeled, and included in the FLUKA (15) simulation code, and will be available for future studies. It will be useful especially in the simulation for the proton therapy, as it will more accurately simulate the production of high linear energy transfer (LET) particles.

ENTERVISION also contributed to the simulation for INSIDE (16), a multimodal monitoring system for the assessment of HT accuracy. One of the researchers developed and benchmarked various FLUKA-based simulations for different scopes. The experimental set-up for a beam test, where prototype detectors and electronics were evaluated, was simulated. The MC prediction was found in good agreement with data, and the code could then be used for the simulation of the full-size detector. Another important aspect was the evaluation, through the simulation of realistic treatment conditions, of the radiation damage induced on the detector by the neutrons produced during patient irradiation. The lifetime of the INSIDE detectors was thus estimated to be at least 5 years. Finally, the specific treatment plan of a patient irradiated at CNAO was simulated using FLUKA, and the results were compared with the commercial TPS used at the facility. The isodose distributions were found in good agreement, and the simulation could then be used to evaluate the Relative Biological Effectiveness (RBE) during treatment.

As prompt gamma monitoring is emerging as a promising imaging modality to monitor the range of the particles used to treat tumors, it is of the utmost importance to have an accurate description of the physical models used in MC tools for modeling the emission of prompt gammas. ENTERVISION performed an extensive and comprehensive analysis of several experiments, in order to create a large set of data to benchmark simulations: these included nine experiments with homogeneous targets such as water and polymethylmethacrylate (PMMA) and three experiments with inhomogeneous targets such as PMMA with a Teflon piece or a lung-equivalent material, performed at several experimental and clinical facilities around Europe and involving different targets, detectors, and set-ups. A real-size prototype for prompt gamma monitoring was developed and optimized, focusing on obtaining the best possible precision in the retrieval of the ion range inside the patient and, at the same time, on providing additional data for comparison with simulations (17).

One of the ENTERVISION researchers participated to an experiment performed in collaboration with University La Sapienza (Rome) where PMMA phantom was irradiated by 220 MeV/u carbon-ions (18). The primary ions outgoing from the exit window were monitored with a plastic scintillator, and two arms were placed at 90° on each side of the phantom. The energy spectra of the prompt-γ produced by interaction of the 12C ions with PMMA target have been measured, and the prompt-γ rates per incident 12C values for the two measured angles were compared and found in agreement. The data were compared with MC simulations performed with Geant4, using two different models: the Quantum Molecular Dynamics (QMD) model of ion-ion collisions and the Binary Cascade light ion model (BIC). An acceptable agreement, both qualitative and quantitative, was obtained between energy spectra (experimental and simulated) and prompt-γ rates, especially for QMD model. Therefore, this study allowed confirming that the QMD model is more accurate than BIC model to reproduce both γ-yields values and γ-spectra as it is the case for charged particles. This originates from the fact that BIC does not take into account properly inelastic scattering processes between ions like (12C + 12C) and (12C + 16O), and also neutron scattering.

ENTERVISION also investigated how graphics processing units (GPUs) can be used to speed up analytical dose calculation for HT (19). Initially, a prototype for a simple dose calculation engine was implemented in Matlab together with a graphical user interface (GUI) and the necessary facilities to open Computed Tomography (CT) images in the Digital Imaging and Communications in Medicine (DICOM) format. The simple dose calculation engine was subsequently implemented to run on GPU and an interface between the GPU code and the GUI was created to allow data to be loaded, stored and analyzed in Matlab, but the calculation to be carried out on a GPU. Following this proof-of-principle study, the work began to create an efficient parallel GPU implementation of the widely used pencil beam algorithm. The implementation was tuned and validated through comparisons between data and MC simulations. The results produced by the GPU implementation showed the same level of accuracy as the dose distribution calculated by the analytical algorithm provided with the commercial TPS used for the treatment. The sub-second calculation times also compared very favorably with those found in the literature, and were short enough to allow for on-line dose calculation applications. Finally, initial work was done to investigate a novel method for analytical dose calculation for proton therapy that would be suitable for parallel implementation.

On the clinical side, weekly 4D CT datasets (9 Non-Small Cell Lung Cancer (NSCLC) patients representing 70 weekly 4D CT datasets) from the University of Texas MD Anderson Cancer Center were used to investigate the impact of several parameters on dose delivery, target coverage and homogeneity, to eventually allow recovery for dose delivery errors caused by intra- and inter-fraction motion. Gating plans (including 4D calculations) were simulated with the GSI treatment planning software TRiP4D (20). Optimization was performed with the first week of each patient using a range-corrected internal target volume (ITV) on states of the moving tumor. The resulting plans were then used for all following weeks. In-depth studies showed that the combination of ITV, isotropic margins, and range margins yielded the best results in terms of target coverage, even though this led to the irradiation of a higher portion of normal tissue. Finally, simulations using one, two, or three fields were performed; for each case, results obtained using ITV only and ITV with additional isotropic and range margins were compared. The best results were obtained using three fields combined to additional isotropic and range margins in terms of target coverage. Using several fields also permitted the reduction of high dose delivery regions in normal tissue. Rescanning will be investigated as a next step to also explicitly address intra-fraction motion. Lung contours extraction is also currently in progress to investigate more precisely the dose delivered to the tissue surrounding the tumor (21).

Finally, ENTERVISION also tackled issues related to biological and physical doses. Development of clinical treatment protocols for any type of cancer RT is dependent on the availability of high quality information on the biological efficacy of radiation doses using a range of beam qualities. This is true especially in HT. In order to gain robust data for use in clinical protocols, multiple cell irradiation experiments must be performed at different dose points, using a range of generic and patient specific tumor cell lines. It is important to be able to verify quickly the biological effects of complex dose distributions in homeomorphic phantoms, alongside measurements of physical dose. A dedicated phantom was designed, tested, and optimized to correctly correlate the biological and physical dose distributions (22).

In this context, specific software for individual cell recognition for microbeam targeting and tracking post-irradiation was developed (23). Bright-field illumination microscopy was chosen as an imaging method in order to avoid potential toxicity from fluorescence excitation. However, the obtained images of cells are characterized by a high degree of complexity since the specialized cell dishes used for microbeam irradiation exhibit highly inhomogeneous optical properties. A cell recognition pipeline has been established using digital image processing techniques and principles from statistics and cluster analysis. This pipeline is able to recognize cellular structures avoiding the majority of the substrate features. It has been tested on both polypropylene and plastic substrates, and in various cell lines including V79 Chinese hamster cells, T98G and U251 human glioblastoma cells.

Additionally, initial time-lapse data have been obtained so as to follow the cells’ life post-irradiation. The biological end-point is the maintenance of cells’ clonogenic ability when irradiated with high-LET radiation using charged particle microbeams. Cell tracking has been applied based on the topological correlation of cells and cell divisions can be effectively detected when cells are separated. Location feedback from frame to frame has been integrated in order to correct false cell detection or linking. The process can be used as a near real-time application in electrostatic cell irradiation. Currently, the software can effectively recognize and irradiate roughly 1,200 cells when real-time tracking is needed, while this number can be increased to more than 2,500 when GPU is used. If real-time tracking is not necessary, then the number of cells capable of irradiation and tracking is only limited by the mechanical properties of the end-station microscope.

### Training Program of ENTERVISION

Network-wide events and training courses were organized throughout the duration of the project. They served the dual purpose of educating the researchers and of creating occasions for them to meet, connect with each other, and establish an extensive professional network with the leading experts in the field.

Courses were aimed at building the researchers’ scientific knowledge, as well as at enhancing their communication and leadership skills (see Table 2). The ENTERVISION technical training portfolio included detectors for medical imaging, electronics, Treatment Delivery Systems, and dosimetry. As health applications need industrial support to be deployed successfully in hospitals and clinics, a course on industrial processes was also run. A course on Intellectual Property management made the young researchers aware of the valorization chain for their scientific results. The ENTERVISION researchers also had the opportunity to join the courses on the impact of gantries and imaging on HT techniques run by a previous Marie Curie Actions Initial Training Network, PARTNER.

**Table 2:** **ENTERVISION network-wide training courses**.

Course title	Trainers and organizers
Basic training ENTERVISION training course: beam production and delivery, hands-on accelerator, treatment planning, dosimetry, radiobiology basics, particle therapy physics	GSI UKL-HD
ENTERVISION Summer School in Lyon: from physics to medical imaging	UCBL CNRS
ENTERVISION Leadership Development Course	UCAM University of Surrey Evolve Leadteam Ltd
Hands-on detectors and electronics course	CSIC IFIC Valencia
Industrial processes	IBA
CV writing	CERN Viva Consult
Intellectual Property	CERN Knowledge Transfer Group

Soft-skills courses tackled leadership, curriculum writing, and communication. The project has been widely disseminated, and the researchers have been encouraged and motivated to take part in outreach activities, at their home institute or elsewhere, including video interviews (24). In September 2013, several ENTERVISION researchers came to CERN to actively participate in the activities for the European Researchers’ night and the laboratory’s Open Days. ENTERVISION also co-sponsored a panel at the EuroScience Open Forum (ESOF) 2014 in Copenhagen chaired by the project coordinator on “Everything you wanted to know about cancer but were afraid to ask.”

The researchers have also been attending the annual meetings of the ENLIGHT network and of the other EC-funded projects run under the ENLIGHT umbrella (in particular of ENVISION). In these occasions, they have presented their work and listened to and interacted with the experts in the HT field, leading to unique learning and networking opportunities.

## Conclusion

ENTERVISION has trained 15 researchers in fields connected to advanced medical imaging techniques for quality assurance during cancer treatment with HT. The researchers have formed a close-knit network, which they are exploiting to their advantage now and for the future. A number of them have already used the contacts they established during ENTERVISION to secure new positions as soon as they finished their Marie Curie projects.

In 2013, ENTERVISION has been chosen as “a success story illustrating the good use of European funds for research” and “as a flagship project for Marie Curie Actions for the promotion of the H2020 program, as a so-called ‘gold project.”’ The EC Directorate-General for Research and Innovation chose 37 projects in total from the previous funding scheme (FP7), with ENTERVISION being the only project representing the Marie Curie Actions. In the same year, ENTERVISION was featured in a press release from the EC to mark the visit to CERN of the EU Commissioner for Education, Culture, Multilingualism and youth.

A number of highly valuable and interesting results have been obtained within the framework of ENTERVISION, as proved by the papers published in this special issue. In addition, 30 posters, 20 oral presentations, and 35 publications featured in international conferences and journals. ENTERVISION researchers took part in the European Researcher’s Night programme and CERN Open days in 2013, and contributed to the publication of Accastampato (25).

## Author Contributions

MD – Project proposer and coordinator of the ENTERVISION and ENVISION projects. MC – ENVISION technical coordinator and overall Communication and Dissemination officer. SN – ENTERVISION dissemination and technical coordinator.

## Conflict of Interest Statement

The authors declare that the research was conducted in the absence of any commercial or financial relationships that could be construed as a potential conflict of interest.
